# Flower evolution of alpine forbs in the open top chambers (OTCs) from the Qinghai-Tibet Plateau

**DOI:** 10.1038/srep10254

**Published:** 2015-05-22

**Authors:** Chan Zhang, Lin-Lin Wang, Yong-Ping Yang, Yuan-Wen Duan

**Affiliations:** 1 Key Laboratory for Plant Diversity and Biogeography of East Asia, Kunming Institute of Botany, Chinese Academy of Sciences, Kunming 650201, Yunnan, P. R. China; 2 Plant Germplasm and Genomics Center, the Germplasm Bank of Wild Species, Kunming Institute of Botany, Chinese Academy of Sciences, Kunming 650201, Yunnan, P. R. China; 3 Institute of Tibetan Plateau Research at Kunming, Kunming Institute of Botany, Chinese Academy of Sciences, Kunming 650201, Yunnan, P. R. China; 4 Graduate University of the Chinese Academy of Sciences, Beijing 100049, Beijing, P. R. China

## Abstract

Effects of global changes on biodiversity have been paid more and more attention world widely, and the open top chambers (OTCs) are the most common tools to study the effects of climatic warming on plant diversity. However, it remains unclear how flowers evolve under environmental changes, which could help us to understand the changes of plant diversity in the OTCs. We compared the insect diversity and pollen:ovule (P/O) ratio of eight outcrossing species with different life histories inside and outside the OTCs on the Qinghai-Tibet Plateau, to examine the effects induced by OTCs on the evolution of floral traits. In the OTCs, P/O ratio decreased in annuals, but increased in perennials, indicating an overall trend toward selfing in annuals. We found that the insect diversity differed significantly inside and outside the OTCS, with decreases of dipteran insects and bees. We concluded that changes of P/O ratio in the studied plant species might result from pollination failure, which might be the results of mismatch between flowering time and pollinator activities. We also suggested annuals might be in a more extinction risk than perennials in OTCs, if strong inbreeding depression occurs in these annual outcrossing plants.

Global changes, especially warming, have been paid more and more attention world widely. The complex ecological consequences resulted from warming could change the biodiversity via altering number^1^ and distribution^2^ of plant species. For example, experimental warming induced by open top chambers (OTCs) caused a 26-36% decrease in species richness on the Qinghai-Tibet Plateau, although this decrease was generally dampened by experimental grazing^3^. Alpine and arctic ecosystems are known to be particularly sensitive to climate warming, and thus many experimental examinations on the effects of climatic warming were performed on alpine and arctic areas^4^,^5^. Experimental warming suggested that flowering phenology of plant species could be advanced or delayed, depending on the flowering time of specific species before or after the peak of the summer heat^6^. These changes in flowering time might induce pollination failure of plant species in the warming plots due to the mismatch between plants and the corresponding pollinators, and the long time pollination failure might drive the evolution of mating systems^7^,^8^,^9^. However, the effects of OTCs on plant mating system have rarely been examined.

Pollen:ovule (P/O) ratio is an important indicator of mating system and P/O ratios per hermaphroditic flowers of outcrossing species were generally higher than those of selfing plants^10^,^11^,^12^. Therefore, changes in P/O ratio might reflect the evolutionary trend in mating system. A recent study suggested that variations in P/O ratio among taxa was mainly driven by pollinator efficiency^13^, which to a certain degree could indicate the variations of pollination visitation frequency among taxa. Additionally, intra-specific variations of P/O ratio were also observed within inflorescences, and these variations were attributed to mating environment^14^,^15^,^16^ and/or resource allocation^17^,^18^, indicating that experiments on changes of P/O ratio from flowers subjected to different treatments should be cautiously treated.

The Qinghai-Tibet Plateau is the largest and highest plateau in the world, and this region is predicted to experience “much greater than average” increases in surface temperature in the future. Studies have found that experimental warming causes large and rapid species loss^3^ and deceases rangeland quality^19^ in this region. In addition, the OTCs in the Qinghai-Tibet Plateau around this station have been established for more than ten years^3^. However, it remains unclear how flowers and the mating system of plant species evolve under environmental changes induced by the OTCs, and these results could help us to understand why some plant species disappeared under experimental warming in the OTCs. In the present study, we compared the insect diversity and P/O ratio in eight outcrossing species with different life histories inside and outside the OTCs, to examine the effects induced by OTCs on the evolution of floral traits. Specifically, we addressed the following questions: 1) what are the differences in the insect diversity between inside and outside the OTCs; 2) what are the differences in the P/O ratio between inside and outside the OTCs? Our results would be of great help to understand the evolutionary trend of mating systems of plant species in the OTCs, and provide a preliminary support on the priority of biodiversity conservation in the changing world.

## Results

**Figure 1:**
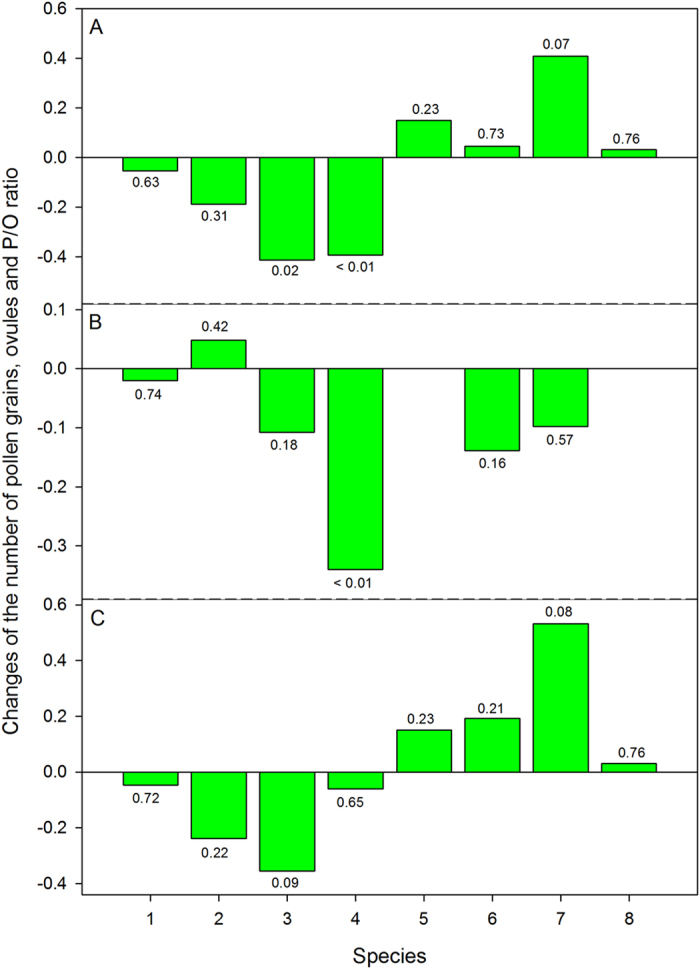
Changes of the number of pollen grains (**A**), ovule (**B**) and pollen:ovule ratio (**C**) in the plants species with different life histories from the Qinghai-Tibet Plateau in the open top chambers. Numbers indicated the comparison results (*P* values) of flowers between the OTCs and control using independent samples *t* test. Species number was shown in Table S1. Plants from No. 1 to 4 are annuals, and from No. 5 to 8 are perennials.

The number of pollen grains of the plant species was affected consistently by the OTCs, specified by the decrease in annual plants and increase in perennial plants, although the difference was not significant between inside and outside the OTCs across all the studied species (Fig. 1A). However, the ovule number did not show consistent changes across the eight plant species (Fig. 1B). Inside the OTCs, the ovule numbers of five plant species decreased, but the ovule number of *Gentiana leucomelaena* increased in the OTCs, and the ovule numbers of *Stellera chamaejasme* and *Allium cyaneum* were constant (Fig. 1B). For the P/O ratio, the effects of OTCs was conflicted for the annual and perennial plants, detailed by the decrease in annual plants and increase in perennial plants (Fig. 1C).

**Figure 2:**
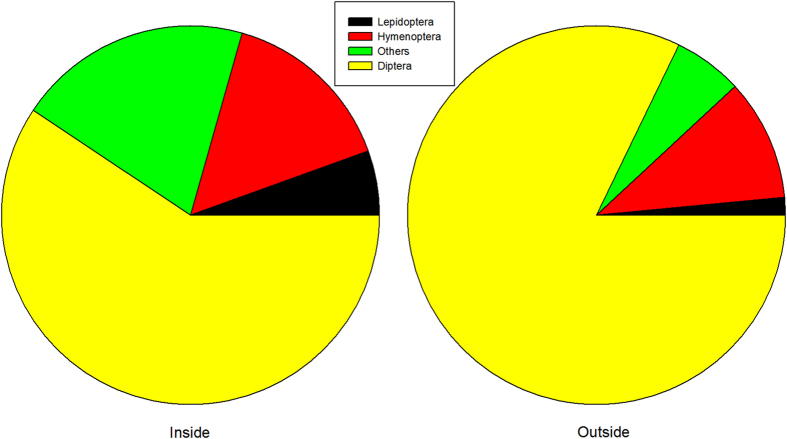
Insect diversity inside and outside the open top chambers.

Overall, our observations suggested that the OTCs changed the insect diversity significantly in comparison with the control outside the OTCs (Fig. 2, χ^2^ = 14.3, *p* = 0.003). Specifically, compared to the control plots, there were ca 4% increases of lepidopteran insects, 4.7% increases of hymenopteran insects, 14.2% increases of other insects and 22.8% decrease of dipteran insects in the OTCs. Furthermore, for the hymenopteran insects, ca 10.3% reduction of the bee visitations were found inside the OTCs in comparison with those outside the OTCs. Collectively, the insect diversity has been changed inside the OTCs, indicating the changed selective pressures on flowers from the pollinators.

## Discussion

For the P/O ratio of our studied species in the OTCs, there was a general decrease in annuals, indicating an evolutionary trend towards selfing. While for the perennials, there was a general increase in P/O ratio in the OTCs, indicating the evolutionary trend towards outcrossing. Variations of P/O ratio were demonstrated recently to be governed by pollination efficiency^13^, and a low P/O ratio is necessary to guarantee seed set, which was generally found in plant species with mating systems of selfing^20^,^21^. Accordingly, changes in P/O ratio could reflect the variations in selective pressures of pollinators and the evolutionary trend in mating system. In the OTCs, we found that insects did not disappear completely, despite of the facts that insect composition inside the OTCs has changed and the dominant pollinators of alpine ecosystems which consisted of bees and flies^22^, have decreased significantly. Thus it seemed that pollinator changes might contribute little to the changes of P/O ratio in the OTCs in our study site. In addition, intra-specific variations of P/O ratio from different positions within inflorescences were common in plant species^14^,^15^,^17^,^18^, but both mating environment hypothesis and resource allocation cannot explain our results since our samples are from the same position on the inflorescences for each species in and outside of the OTCs. It has been demonstrated that experimental warming could advance or delay the flowering time of plant species^6^, which could induce the mismatch between plants and the corresponding pollinators and further reduce pollinator service of plant species in the OTCs. Collectively, changes of P/O ratio of the eight outcrossing plants in the OTCs could be induced by the pollination failure, although this argument needs future experimental supports.

Our results suggested the different responses for annual and perennial plants in the OTCs. There was a trend towards decreased ovule number, but no consistent response was found in the changes in both annuals and perennials (Fig. 1B). However, for the number of pollen grains, we found that there was a decreasing trend for the four annual plants and an increasing trend for the perennial plants (Fig. 1A), suggesting that the variations in P/O ratio of plants could be resultant from changes in the number of pollen grains, and indicating that resource allocation to male functions of plant species were more susceptible to environmental changes^23^. Importantly, different evolutionary trends in P/O ratio of plants with different life histories could be explained in the following. For the annual plants, long-time pollination failure might drive the evolution of selfing, and then reduced P/O ratio was expected^10^,^13^. However, for the perennials, because reproduction is costly in plants, current failures in seed production might induce the enhanced investments to future reproduction under the hypothesis on the trade-off between current and future reproduction^24^. Furthermore, higher cost of reproduction in females than in males^25^ could drive the future allocation to pollen production, resulting in the increased P/O ratio of the perennials in the OTCs. Therefore, the life history traits could contribute to the different responses in P/O ratio of plants since the ten-year’s treatment is far less than the history of angiosperm, although evolutionary trends were obvious in both annuals and perennials.

The temperature inside the OTCs has been elevated across the growing seasons^26^, but the evolutionary trend towards selfing in annual plants could not be resultant from experimental warming. Furthermore, this evolutionary trend might increase the extinction risk of annual plants with mating system of outcrossing, if selfing occurs in these species and inbreeding depression is strong^27^. Therefore, facing the global change, especially global warming, we tentatively suggested that we should give a conservation priority to those endangered annual plants with outcrossing, indicating the importance of studying breeding systems of endangered and endemic annual plants in the future conservation of biodiversity. Furthermore, we tentatively draw another conclusion that the widely used OTCs for experimental warming might not be an ideal method in studying the effects of warming on special level, especially when studying the relationships between plants and their mutualisms, e.g. pollinators, florivores, herbivores and seed predators.

## Materials and Methods

### Study site and materials

Our studies were carried around the Haibei Alpine Meadow Ecosystem Research Station, the Chinese Academy of Science. The station is located on the northeast corner of the Qinghai-Tibet Plateau, with lat. 37°29′-37°45′ N, long. 101°12′-101°23′ E, and alt. 3200 m. The average annual air temperature is −1.7 °C with extremes of 27.6 °C (maximum) and −37.1 °C (minimum). Average annual precipitation ranges from 426 to 860 mm, 80% of which falls in the summer from late May to early September^28^.

The open top chambers (OTCs) around this station have been established for more than ten years^3^. The OTCs, which were 1.5 m diameter and 40 cm height, were constructed of Sun-Lite HP (Solar Components Corporation, Manchester, NH, USA) 1.0 mm thick fiberglass and remained on the grassland year-round^3^. The OTCs consistently elevated growing season averaged mean daily air temperature by 1.0–2.0 °C, maximum daily air temperature by 2.1–7.3 °C and the diurnal air temperature range by 1.9–6.5 °C, with mixed effects on minimum daily air temperature, and mean daily soil temperature and moisture^26^.

The eight studied species and their characteristics are listed in Table S1, and all of the eight species are the common forbs inside and outside the open top chambers (OTCs). According to our observations, netting treatment and former studies^29^,^30^,^31^,^32^, flowers of the eight plants cannot produce seeds without aids of pollinator, indicating (facultative) outcrossing mating systems in the eight plants. For example, all collected plant materials in Gentianaceae in the experiments are characteristics of protandry and/or herkogamy.

### Methods

#### Pollen and ovules

For each of the eight species (Table S1), we selected twenty flower buds that will be open shortly inside and outside the OTCs, and fixed them in standard FAA solution (formalin:acetic acid:100% ethanol at a ratio of 5:5:90 by volume). To avoid the effects of architecture and resources from different positions on flower traits^33^, only the terminal flowers were selected in this experiment. In the laboratory, We squashed all anthers from each flower and suspended all pollen grains in 5 mL of water with drops of detergent solution for full suspension^34^. Total pollen in each flower was determined by counting the number of pollen grains in 20 drops of pollen solution (5 μL) with a light microscope. The number of ovules was determined under a stereoscope by dissecting all the carpels of the flower. We then calculated the pollen:ovule ratio for each flower of each species.

#### Insect observations

In the late May, 2011, we established ten plots adjacent to the OTCs, and each plot was similar to the OTC in size, and observed insects that entered the OTCs and the plots from early June to late August. In each month of the early-, middle- and late-stage, we monitored and recorded the insects in three sunny days without strong wind from 9:30 to 16:30 when the temperature was high, and we would cease the observations if rain occurred. Totally, the observation time amounted to 170 h. In light of our long time observations and experiments on pollination ecology of alpine plant around this station^35^, the insects were classified as Lepidopteran, Hymenopteran, Dipteran and other insects, such as beetles.

#### Statistical analysis

The difference in the insect diversity inside and outside the OTCs were analyzed using a Chi-square test, and the number of pollen grains and ovules, and pollen:ovule ratio were ln-transformed before comparison, and independent sample *t* test was used to compare the differences inside and outside OTCs. The effects of OTCs were calculated as ln(X_T_/X_C_), where X_T_ and X_C_ are the averaged data of flowers in OTCs and control (outside OTCs), respectively^36^.

## Author Contributions

Y.W.D. and Y.P.Y. designed the research and wrote the manuscript; C.Z., L.L.W. and Y.W.D performed experiments; Y.W.D analysed data and prepared the figures and tables.

## Additional Information

**How to cite this article**: Zhang, C. *et al*. Flower evolution of alpine forbs in the open top chambers (OTCs) from the Qinghai-Tibet Plateau. *Sci. Rep.* **5**, 10254; doi: 10.1038/srep10254 (2015).

## Supplementary Material

Supplementary Information
