# Supplementary material for: Flower evolution of alpine forbs in the open top chambers (OTCs) from the Qinghai-Tibet Plateau
Source: Sci Rep. 2015 May 22;5:10254. doi: 10.1038/srep10254 (PMC4441194; doi:10.1038/srep10254)
Supplement: Supplementary Information [file srep10254-s1.pdf]

Title: Flower evolution of alpine forbs in the open top chambers (OTCs) from the Qinghai-Tibet Plateau

Authors: Chan Zhang, Lin-Lin Wang, Yong-Ping Yang, Yuan-Wen Duan

Supplementary Table S1 Traits of eight outcrossing plant species and their flowers. The ovule numbers of species 5 and 8 are constant.

No.	Species name	Family	LH	Pollen number		Ovule number		Pollen:ovule ratio	
				C	OTC	C	OTC	C	OTC
1	*Euphrasia pectinata* Ten	Scrophulariaceae	A	1161 ± 93	1100 ± 84	30 ± 1	30 ± 1	39 ± 4	37 ± 3
2	*Gentiana leucomelaena* Maxim	Gentianaceae	A	10089 ± 1374	8356 ± 569	139 ± 5	146 ± 9	74 ± 10	58 ± 4
3	*Gentianella azurea* (Bunge) Holub	Gentianaceae	A	6560 ± 592	4340 ±649	45 ± 3	41 ± 2	155 ± 20	108 ± 16
4	*Lomastogonium carinthiacum* (Wulf.) Reichb	Gentianaceae	A	24000 ± 1484	16216 ± 1314	346 ± 35	246 ± 20	73 ± 6	69 ± 7
5	*Stellera chamaejasme* L.	Thymelaeaceae	P	9520 ± 838	11066 ± 918	1	1	9520 ± 838	11066 ± 918
6	*Oxytropis kansuensis* Bunge	Leguminosae	P	13204 ± 1450	13808 ± 932	12 ± 1	10 ± 1	1182 ± 147	1434 ± 126
7	*Gentiana straminea* Maxim	Gentianaceae	P	52955 ± 4951	79660 ± 2221	235 ± 26	213 ± 27	249 ± 33	423 ± 84
8	*Allium cyaneum* Regel	Liliaceae	P	34560 ± 2522	35664 ± 2560	6	6	5760 ± 420	5944 ± 427

LH, life history; A = annual, P = perennial. Data are shown as Mean ± 1 SE. C, control flowers; OTC, flowers in OTCs.
